# Plasma exosomes exacerbate alcohol- and acetaminophen-induced toxicity via CYP2E1 pathway

**DOI:** 10.1038/s41598-019-43064-2

**Published:** 2019-04-25

**Authors:** Mohammad A. Rahman, Sunitha Kodidela, Namita Sinha, Sanjana Haque, Pradeep K. Shukla, Radhakrishna Rao, Santosh Kumar

**Affiliations:** 1 Department of Pharmaceutical Sciences, College of Pharmacy, University of Tennessee Health Science Center, Memphis, TN 38163 USA; 2 Department of Physiology, University of Tennessee Health Science Center, Memphis, TN 38163 USA

**Keywords:** Biochemistry, Enzymes

## Abstract

Cellular CYP2E1 is well-known to mediate alcohol- (ALC) and acetaminophen- (APAP) induced toxicity in hepatic and extra-hepatic cells. Although exosomes have been gaining importance in understanding mechanism of intra- and inter-cellular communication, the functional role of drug metabolizing cytochrome P450 (CYP) enzymes in human plasma exosomes are yet to be explored. In our previous study, we reported that human plasma-derived exosomes contain substantial level of functional CYP2E1. In the current project, we investigated the potential role of plasma exosomal CYP2E1 in mediating ALC- and APAP-induced toxicity. We treated hepatic and extra-hepatic (monocytic) cells with exosomes ± ALC/APAP. We observed that the plasma exosomes containing CYP2E1 cargo further exacerbate ALC- and APAP-induced toxicity in both hepatic and monocytic cells. Further, both exosomes- and ALC/APAP-induced toxicity was reduced/abolished by a selective inhibitor of CYP2E1 enzyme activity (diallyl ether). However, only ALC-, but not exosome-induced toxicity was reduced/abolished by CYP2E1 siRNA. These findings suggest that ALC/APAP-induced toxicity in the presence of exosomes are mediated, at least in part, by CYP2E1 enzyme. To validate these *in vitro* findings, we characterized plasma exosomal contents in a binge-drinking animal model and their effect on ALC/APAP-induced toxicity in monocytic cells. Our results showed that ALC exposure caused a significant induction of the plasma exosomal CYP2E1 level in a binge drinking murine model. These exosomes containing increased levels of CYP2E1 caused significant toxicity in monocytic cells compared to exosomes derived from control mice. Overall, our results showed an important role of exosomal CYP2E1 in exacerbating ALC- and APAP-induced toxicity. The study is significant in terms of understanding the role of exosomal CYP2E1 in cell-cell interactions, and their effects on drug-induced toxicity.

## Introduction

Cytochrome P450 (CYP) enzymes are key components in drug metabolism and xenobiotic- induced toxicity. At high doses, oxidative stress and formation of toxic metabolites mediated by CYP2E1 are known as the principal pathway of toxicity induced by alcohol (ALC) and acetaminophen (APAP)^1–4^. Ethanol-induced oxidative stress is not restricted to the hepatic cells, where ethanol is actively oxidized, but can affect various extra-hepatic tissues^5–7^. Since CYP2E1 is expressed in lower magnitude in the extra-hepatic regions than in the liver, the drug overdose-associated toxicity is not always clearly understood in extra-hepatic cells. Previously, we have shown that alcohol exposure increases human immunodeficiency virus (HIV) replication in monocytic cells via CYP2E1-mediated oxidative stress^8,9^. However, the exact mechanism of alcohol-exacerbated HIV replication in monocytic cells via CY2E1-mediated oxidative stress is not clear.

Literatures suggest a potential contribution of exosomes as a key modulator in xenobiotics- and disease-induced toxicity in various tissue systems^10,11^. Exosomes, the small extracellular nanovesicles produced by most cell types, are emerging as a novel tool for investigation of disease pathogenesis and in drug discovery research^12–16^. Exosomes can play a vital role in intra- and inter-cellular communication via selective packaging and transport of microRNAs (miRNAs), mRNAs, proteins, and other biological cargos to neighboring cells or to distant tissues via biofluids such as plasma, urine, cerebrospinal fluid, etc.^17–21^. Their unique ability to navigate through the biological system, transport important biomolecules, and influence the pathophysiology in the recipient cells make exosomes an attractive candidate for biological and biopharmaceutical research^22–24^. Recently, plasma exosomes have been studied extensively to discover reliable biomarkers for diseases such as cancer, kidney injury etc.^24–27^. Exosomes in plasma are secreted from a variety of tissues, and therefore, they are an excellent resource for examination of disease pathogenesis.

The presence of some of the most physiologically relevant CYP enzymes, including CYP2E1, in human plasma exosomes was recently reported by our group^28^. We observed that exosomes derived from plasma of healthy subjects packaged metabolically active CYP2E1 as well as other CYP isoforms. Interestingly, the level of CYP2E1 mRNA and protein level in plasma exosomes were significantly higher than the other CYPs such as CYP3A4. A recent report by our group demonstrated that there are significant differences in the packaging of inflammatory cytokines (e.g. interleukins- 6, 8, 10) in plasma exosomes isolated from HIV-infected ALC drinker^29^. Exposure of ALC or APAP to hepatic cells induces CYP2E1 and potentially increases packaging of CYP2E1 in exosomes^6,30,31^. Therefore, we hypothesized that these exosomes can transfer CYP2E1 to naïve hepatic and extra-hepatic tissues via biological fluids and mediate ALC- and APAP-induced toxicity.

In this study, we examined whether plasma exosomal CYP2E1 cargo play a role in exacerbating ALC- and APAP-induced toxicity in hepatic and extra-hepatic cells.

## Results

### Identification and characterization of plasma exosomes

**Figure 1:**
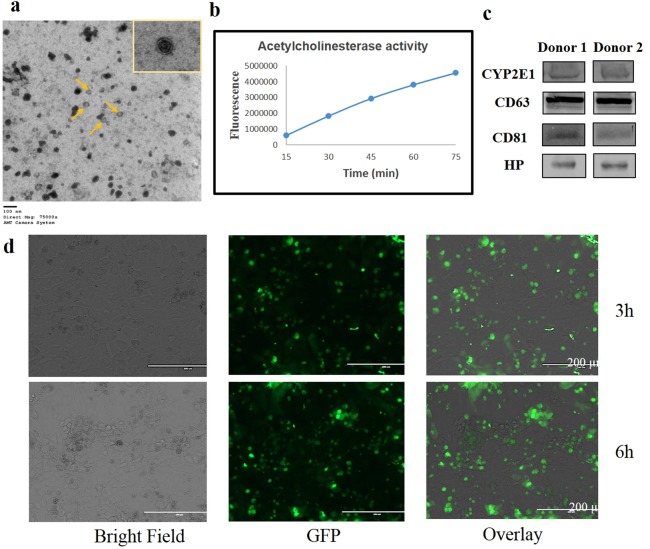
Identification and uptake of exosomes derived from human plasma. (**a**) Transmission electron microscope (TEM) images of exosomes (arrows indicate exosomes). (**b**) Acetylcholinesterase activity in exosomes. (**c**) Representative immunoblots for the expression of exosomal marker protein CD63 and CD81, and liver specific exosomal protein HP in plasma exosomes. Equal amount of protein (10 μg) was loaded in each lane. (**d**) Exosome uptake in HepaRG cells. Bright field, fluorescent, and overlay images of cells after 3 and 6 hours of exosomes exposure. Microscope was set to visualize particles of ≤200 μm diameter. Bright field and fluorescent images represent only cells and cells with exosomes, respectively. Overlay images demonstrate the presence of exosomes within the cells. GFP-labelled green circles represent accumulation of exosomes internalized by the cells.

We isolated exosomes from healthy human plasma as previously described and characterized their physicochemical properties^28,32^. Transmission electron microscopy (TEM) images confirmed the presence of exosomes (double-membraned vesicles) in the prepared sample (Fig. 1a). Further, we showed a time-dependent increase in acetylcholine esterase activity (Fig. 1b) and the presence of CD63 and CD81 (Fig. 1c), which are specific markers for exosomes, confirming the identification of exosomes. Further, we showed the presence of haptoglobin (HP) (Fig. 1c), a specific marker protein for hepatocyte-derived exosomes, suggesting the presence of liver-derived exosomes in the plasma. Since liver is the powerhouse of CYP enzymes, it is likely that the majority of the CYP enzymes in the plasma exosomes are secreted from the liver. Moreover, CYP enzymes in plasma exosomes may come from a variety of other cells because CYPs are known to be expressed in extra-hepatic tissues/cells such as lung, kidney, etc. Previously, we have shown that plasma exosomes contain substantial amount of CYP2E1 mRNA and protein in healthy human donor^28^. In this study, we also found that plasma exosomes from two healthy donors contain significant level of CYP2E1 protein (Fig. 1c).

### Uptake of exosomes in the recipient cells

To determine whether the exosomes are involved in cell-cell communication, we performed an exosome uptake assay. In this study, we used HepaRG hepatocytes and the HIV-1-infected monocytic U1 cell line. HepaRG cells are considered as a reliable *in vitro* model for studying hepatic drug metabolism and liver-related pathologies. They express the majority of phase I and II metabolic enzymes, including CYP2E1, and CYP2E1 is further induced by ALC in HepaRG cells^33–35^. Similarly, among extra-hepatic cells, HIV-infected monocytic cells (U1) express cellular CYP enzymes, especially CYP2E1, which is also induced by ALC exposures^6,8^. We exposed plasma exosomes to HepaRG cells. As shown previously^32^ with U1 cells, our fluorescence microscopy imaging showed that exosomes are readily taken up by the recipient hepatocytes after 3–6 hours of exposure (Fig. 1d).

### Effect of human plasma exosomes on hepatocytes upon ALC and APAP exposure

**Figure 2:**
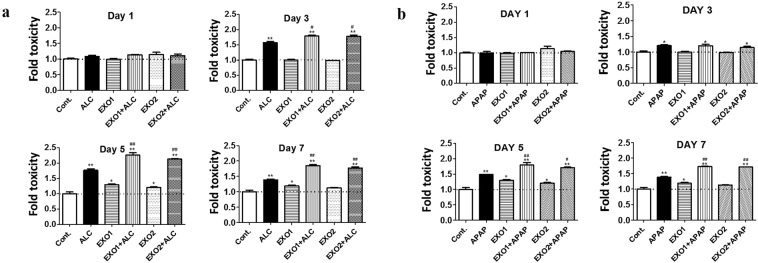
(**a**) Effect of plasma exosomal CYP2E1 in HepaRG cells upon ALC treatment. Plasma exosomes were isolated from healthy human plasma and then treated to HepaRG cells ± alcohol (ALC). Exosome 1 (EXO1) and EXO2 represent exosomes isolated from two different volunteers. Cytotoxicity was measured every day using LDH cell viability assay. The data shown here represent the average of 3 experiments. One-way ANOVA was used to measure statistical significance. *Represents significance when compared to control, ^#^represents significance when compared to ALC. (**b**) Effect of plasma exosomal CYP2E1 in HepaRG cells upon APAP treatment. Plasma exosomes were isolated from healthy human plasma and then treated to HepaRG cells ± APAP. EXO1 and EXO2 represent exosomes isolated from two different volunteers. Cytotoxicity was measured every day using LDH cell viability assay. The data shown here represent n#3 experiments. One-way ANOVA was used to measure statistical significance. *Represents significance when compared to control, ^#^represents significance when compared to APAP.

In our previous study, we observed that plasma exosomes contain large amounts of CYP2E1 enzyme relative to hepatocytes or hepatocyte-derived exosomes^28^. To investigate the effect of plasma exosomal CYP2E1, we co-treated HepaRG cells with exosomes (derived from 50 µL of clarified plasma) along with 50 mM ALC and 0.5 mM APAP. As expected, both ALC and APAP showed time-dependent increase in toxicity in HepaRG cells, which are known to be mediated through CYP2E1 pathway. We further observed that treatment with plasma exosomes caused a significant increase in ALC-induced toxicity in a time-dependent manner (Fig. 2a). Similarly, plasma exosome exposure resulted in increased APAP-induced toxicity (Fig. 2b). These results suggested that CYP2E1 cargo in plasma exosomes may contribute to the toxicity induced by ALC and APAP. It is important to note that plasma exosomes treatment alone caused significant toxicity from 5^th^ day onwards, perhaps due to increased metabolism of endogenous substrates as well as delivery of oxidative stress related miRNA (e.g. mir200 family) resulting in enhanced oxidative stress and cytotoxicity.

### The role of plasma exosomal CYP2E1 in mediating ALC- and APAP-induced toxicity

**Figure 3:**
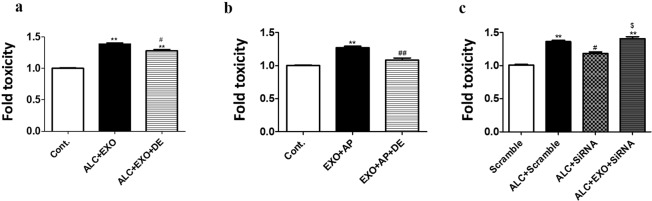
Rescue of exosomal CYP2E1 mediated toxicity by selective CYP2E1 inhibitor (DE) and CYP2E1 siRNA. (**a**,**b**) HepaRG cells were treated with human plasma exosomes ± ALC/APAP with/without DE for 2 days. One-way ANOVA was used to measure statistical significance. *Represents significance when compared to control, ^#^compared to EXO + ETH/APAP. (**c**) HepaRG cells were treated with human plasma exosomes ± ALC with/without CYP2E1 siRNA. Cytotoxicity was measured using LDH cell viability assay. The data shown here represent n#3 experiments. One-way ANOVA with Tukey’s multiple comparison test was used to measure statistical significance. ^#^Represents significance when compared against scramble, ^$^compared to ALC + scramble, ^compared to ALC + si2E1.

To examine whether plasma exosomal CYP2E1 contributes to ALC- and APAP-induced toxicity, we pretreated the hepatic cells with selective CYP2E1 inhibitor and siRNA. In our laboratory, we have identified several analogs of diallyl sulfide (DAS) as more potent and relatively safer CYP2E1 inhibitors. Diallyl ether (DE) is one of the analogs with the best CYP2E1 inhibitory capacity and lowest toxicity profile^36,37^. We pretreated HepaRG cells with ALC and/or APAP and plasma exosomes with or without pre-treatment with DE. In both set of treatments, DE significantly reduced the additional toxicity caused by co-treatment of ALC/APAP and plasma exosomes (Fig. 3a,b). To further confirm the potential impact of plasma exosomal CYP2E1 in ALC-induced toxicity, we silenced the cellular expression of CYP2E1 in HepaRG hepatocytes with CYP2E1 siRNA and then exposed cells with ALC + exosomes. In principle, SiRNA should not inhibit exosomal CYP2E1 enzymes. As expected, we observed that CYP2E1 siRNA treatment significantly rescued the cells from ALC-induced toxicity, while it did not reduce/abolish exosome-induced toxicity (Fig. 3c). The data clearly suggests that the exacerbation of ALC/APAP-induced toxicity by plasma exosomes is mediated by delivery of exosomal CYP2E1.

### Effect of human plasma exosomes on U1 monocytic cells

**Figure 4:**
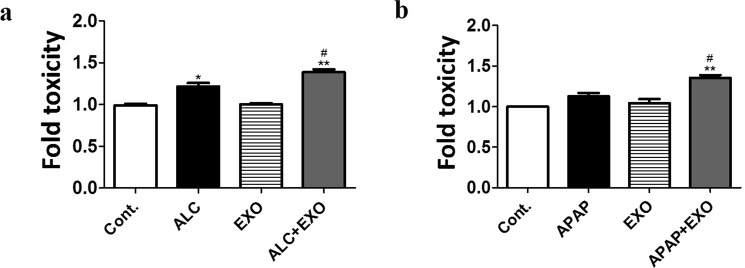
Effect of plasma exosomal CYP2E1 in U1 cells upon ethanol treatment. Plasma exosomes were isolated from healthy human plasma and then treated to U1 cells +/− ALC/APAP for 2 days. Cytotoxicity was measured using LDH cell viability assay. The data shown here represent n#3 experiments. One-way ANOVA was used to measure statistical significance. *Represents significance when compared to control, ^#^compared to ALC/APAP.

We previously reported that ALC-induced CYP2E1 expression in monocytic/macrophage cell lines resulted in increased oxidative stress and cytotoxicity^8^. It is likely that the addition of external CYP2E1 load into an *in vitro* system will exacerbate CYP2E1-mediated toxicity in U1 cells. Therefore, we treated U1 cells with 50 mM ALC (physiological concentration of ethanol in binge drinking^38^) and 0.5 mM APAP (sub-toxic physiological concentration^39^) along with plasma exosomes (obtained from 50 µL plasma). After 2 days, we observed that plasma exosome treatment caused increased toxicity with both ALC and APAP co-treatment (Fig. 4a,b). This result further suggests that plasma exosomal CYP2E1 cargo can have significant physiological effects not only in hepatic, but in extra-hepatic cells as well.

### Effect of alcohol on exosomal CYP2E1 derived from mice plasma

**Figure 5:**
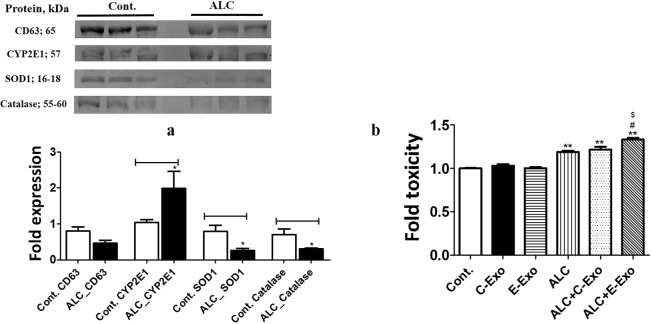
Effect of alcohol on murine plasma exosomal CYP2E1 and AOEs. (**a**) Expression of CYP2E1, SOD1, and catalase in plasma exosomes in binge-drinking mice. (**b**) Effect of exosomes derived from ALC fed mice on ALC-induced toxicity in U1 cells after 4 days. One-way ANOVA was used to measure statistical significance. *Represents significance compared to control and control-exosomes (C-Exo), ^#^represents significance compared to ALC induced exosomes (E-Exo), ^$^represents significance relative to ALC and ALC + C-Exo. Full-length blots/gels are presented in Supplementary Fig. 1.

To corroborate our *in vitro* findings, we isolated exosomes from the plasma of mice treated with and without alcohol (binge exposure). Although the role of CYP2E1 in alcohol-induced liver toxicity is well known in alcohol-drinking mice model, the contribution of plasma exosomes via CYP2E1 in toxicity is unknown. Therefore, we measured changes in the protein expression of CYP2E1 and two antioxidant enzymes (AOEs), SOD1 and catalase along with exosomal marker protein CD63 (Fig. 5a). Interestingly, the average level of CD63 was reduced in alcohol-treated mice. The most crucial observation, however, was an increase in the level of CYP2E1 in binge-drinking mice (Fig. 5a). It can be noted that this change in CYP2E1 was obtained without normalizing the CYP2E1 band against the exosomal marker protein. The levels of the AOEs, SOD1 and catalase, were also decreased, suggesting an overall increase in oxidative stress components in the exosomes derived from ALC-treated mice.

### Effect of mouse plasma exosomes on U1 cells upon ethanol treatment

Since we observed increased CYP2E1 and reduced AOE expression in exosomes derived from ALC-treated mice, we anticipated that this phenomenon will contribute to enhanced ALC-induced toxicity in the recipient cells. To examine this, we co-treated U1 cells with 50 mM ALC ± exosomes isolated from control or binge-drinking mice. After 4 days of ALC treatment, we observed that exosomes derived from ALC-exposed mice caused significantly higher toxicity than exosomes from the control group (Fig. 5b).

## Discussion

CYP2E1, one of the key components of the microsomal ethanol oxidizing system (MEOS), is mainly involved in the metabolism of small molecular xenobiotics^40^. CYP2E1 can activate procarcinogens and other toxic compounds found in cigarette smoke and various environmental pollutants^41–43^. However, the most clinically relevant role of CYP2E1 is associated with ALC and APAP metabolism, especially at their higher doses. Though liver is the primary site where CYP2E1-mediated metabolism exerts its most damaging effect, extra-hepatic tissues are often affected to a various degree as well^44,45^. While the hepatic pathway of ALC- and APAP-induced toxicity is well established, the extra-hepatic mechanism(s) for ALC- and APAP-induced toxicity is not clearly defined. We recently reported that CYP2E1 is abundantly present in exosomes derived from healthy human plasma^28^. We also predicted that the majority of the CYP2E1 in plasma exosomes are derived from hepatic cells, along with some from extra-hepatic cells. Based on our previous findings, we hypothesized that the metabolically active plasma exosomal CYP2E1 cargo participates in cellular pathophysiology and contributes to ALC- and APAP-induced toxicity in hepatic and non-hepatic cells.

**Figure 6:**
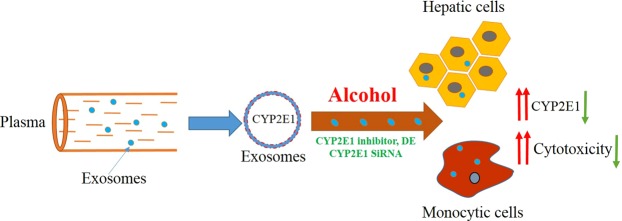
Schematic representation of the plasma exosomal CYP2E1 mediated toxicity. Plasma derived exosomes can carry metabolically active CYP2E1 which may influence the metabolic pathway of ALC and APAP in hepatic and extra-hepatic cells resulting in increased cytotoxicity.

In our current study, we observed that plasma exosomes treatment additively/synergistically increased ALC- and APAP-induced toxicity in HepaRG hepatocytes and U1 monocytic cells. Further, using CYP2E1 specific inhibitor and CYP2E1 siRNA, we confirmed the role of CYP2E1 in exosomes induced toxicity. Besides, we found that alcohol exposure increases packaging of CYP2E1 in exosomes derived from plasma of mice. We also observed enhanced toxicity when these ALC-induced exosomes were treated to U1 cells, compared to treatment with exosomes derived from non-drinking mouse plasma. The results suggest that the exosomal cargos, specifically CYP2E1, may have far-reaching metabolic consequence in ALC- and APAP-induced pathogenesis in hepatic and extra-hepatic cells (Fig. 6).

Exosomes are released by almost all cell types into their extracellular space or into biofluids such as plasma^46^. Recent studies on exosomes reveal that they can be actively involved in intercellular communication with both beneficial and harmful physiological consequences^32,47–49^. Exosomes have been suggested as a potential biomarker in ALC and APAP associated pathologies. Several studies have reported circulating exosomal miRNAs as potential markers for ALC- and other drug-induced inflammatory liver diseases. For example, Momen-Heravi *et al*., (2015) showed that liver derived exosomal transfer of miR-122 rendered monocytes more sensitive to inflammation, which is a key phenomenon in AH pathogenesis^50^. They also showed that the number of exosomal production was increased and the ethanol concentration in the human sera was reduced after ALC consumption. Another study demonstrated that ALC mediated increased EV production and macrophage activation follows CYP2E1-dependent pathway^51^. Similar studies have been conducted relating exosomal pathway and APAP-induced injury. Cho *et al*., (2018) showed that exosomes obtained from APAP-induced liver injury caused toxicity in the naïve recipient hepatocytes in a mouse model^49^. Another study suggests that alteration in the hepatocytes derived exosome levels can act as an early predictor of liver injury in subtoxic APAP exposure^52^. These studies are shedding new light in the phenomenon of xenobiotics-induced toxicity and disease pathogenesis.

Exosomal miRNAs, especially the mir200 family, have been hypothesized as a major target to combat oxidative stress-induced effects^53^. CYP2E1-mediated increased oxidative stress in hepatocytes upon ALC exposure is expected to induce mir200s, which are packaged in exosomes, secreted, and circulated in the plasma^54^. The mir200s promote oxidative stress by disrupting the SIRT1/FOXO1/eNOS pathway and inhibiting transcription of AOEs, especially catalase^55^. Dysregulation of mir-200s and resultant oxidative stress cause apoptosis and cellular senescence in many cells^56^. Hence, exosomal mir200s- and CYP2E1-induced oxidative stress would enhance ALC-induced toxicity additively and/or synergistically. We have observed that plasma exosomes derived from alcohol drinker subjects contain significant level of mir-200c-3p compared to healthy subjects (unpublished observation).

In this project, we observed a somewhat similar phenomenon when plasma exosomes from a binge-drinking mouse model were characterized for their AOE expression. The expression of CYP2E1 enzyme was significantly upregulated, while SOD1 and catalase were downregulated upon alcohol exposure in these mice. When these exosomes were treated or exposed to recipient U1 monocytic cells, the ALC-induced toxicity was further exacerbated. The overall increase in oxidative stress elements mediated by CYP2E1 in the ALC-induced exosomes may have acted as an underlying factor in the increased toxicity.

Through the findings of this study we suggest that plasma exosomes contribute to ALC/APAP-induced toxicity, via delivery of CYP2E1 to cells that have high cellular CYP2E1 (hepatic) as well as low cellular CYP2E1 (monocytic) levels. Upon further validation, the plasma exosomal CYP2E1 pathway may help to better understand ALC-, APAP-, and other xenobiotic-induced toxicity, especially in the extra-hepatic tissues. Plasma exosomes as “liquid biopsy” has been gaining attention in the drug metabolism and drug- drug interaction (DDI) arena of drug development^57^. Improvement of analytical techniques has enabled the quantification of many endogenous components in human biofluids such as plasma. Hence, plasma exosomal CYP2E1 load, along with other relevant CYPs, can be taken into consideration while studying DDI and pharmacokinetic profiling of new and existing drugs. This will not only help understanding drug-induced pathologies in the hepatic and extra-hepatic tissues but also in optimizing dosing regimens.

In conclusion, this study demonstrates that both human and murine plasma exosomal cargo, specifically CYP2E1, play a role in exacerbating ALC- and APAP-induced toxicity in hepatocytes and monocytes. Exosomal transport between cells can be either beneficial or harmful, and thus a better understanding of the roles of exosomes in drug abuse/overdose conditions is crucial. Further studies, especially using human plasma exosomes from alcohol drinkers are required to explore the full potential of the plasma exosomal cargo, especially CYP2E1 and other CYP enzymes, as biochemical mediators in drug metabolism and resulting toxicity. Specific and relative contributions as well as underlying mechanism of hepatic- and extra-hepatic-derived exosomes in circulating plasma is also crucial for further studies.

## Methods

### Chemicals

Acetaminophen (catalog #A5000-100G) and diallyl ether (purity 98%, catalog # 259470) were purchased from Sigma-Aldrich (St. Louis, MO). Two hundred proof ethanol (catalog #A405P4) was purchased from Fisher Scientific (Hampton, NH).

### Cell culture and treatment

For the current study, two types of cell lines were used: (1) HepaRG hepatocytes and (2) latently HIV-1-infected monocytic U1 cells. Terminally differentiated HepaRG cells (ThermoFisher Scientific, Grand Island, NY) are the most reliable and appropriate cell lines for hepatocyte research due to their similar expression profile of CYPs and other metabolic enzymes when compared with primary hepatocytes^58^. The U1 cells (NIH AIDS Reagent Program; Germantown, MD) are recognized as the model system for investigating HIV effects in monocytes^59^. HepaRG cells were cultured in media consisting of William’s E Medium supplemented with HepaRG Tox/Maintenance Medium Supplement^®^ and Glutamax. First, the cells were plated using maintenance media. After 24 hours, maintenance media was replaced with tox media and cells were allowed to reconstitute in monolayer. The treatment was started at the 7^th^ day since the peak CYP expression occurs from days 7–10. The U1 cells were cultured in Roswell Park Memorial Institute (RPMI) 1640 media (Sigma Aldrich, St. Louis, MO) supplemented with 10% fetal bovine serum, L-glutamine, sodium bicarbonate, non-essential amino acids, and penicillin-streptomycin solution. The cells were maintained in a humidified incubator with 5% CO_2_ at 37 °C. Cells were co-treated with either 50 mM ethanol or 0.5 mM acetaminophen along with isolated human or mouse plasma exosomes for 1–8 days. We used the CYP2E1 inhibitor diallyl ether (20 µM) to validate the role of plasma exosomal CYP2E1 in ALC- or APAP-induced toxicity. Cell culture media was collected for use in the LDH cell viability assay and replaced with an equivalent amount of fresh media. At the end of treatment, cells were harvested for performing protein extraction and western blot assay.

### Isolation and validation of exosomes from human and mouse plasma

Exosomes were isolated from both human and mouse plasma for this project. Human blood samples were collected from de-identified healthy individuals at Interstate Blood Inc. (Memphis, TN). Exosomes were isolated using the Plasma Exo Kit (Applied Biosystems, Foster City, CA). In brief, a 0.22 µm filter was used to remove larger vesicles (>200 nm) from the plasma. Afterwards, the human and mouse samples were centrifuged at 2,000 and 10,000 g, respectively for 20 min to remove cell debris. To the clarified plasma, 0.5 volume of 1X PBS was added and mixed thoroughly. Afterwards, 0.2 volume of exosome precipitation reagent was added, and the samples were mixed well by vortexing. After a 10 min incubation at room temperature, samples were centrifuged at 10,000 g for 5 min. The resulting pellet was resuspended using appropriate medium and used for downstream procedures. We checked for expression of the standard exosomal marker proteins such as CD63 and CD81 by western blotting. We also tested whether plasma exosomes contained a liver-specific HP protein, since the liver is one of the major contributors of plasma exosomal CYP enzymes. Exosomes were also validated in terms of size, shape, and quality by using Transmission Electron Microscopy, following a standard protocol as described previously^32^ (JEOL 2000EXII TEM, Neuroscience Institute, The University of Tennessee Health Science Center).

### Acetylcholine esterase assay

The quality of the isolated exosomes was further verified by measuring acetylcholinesterase activity using the validated Amplex^TM^ Red Acetylcholine/Acetylcholinesterase Assay Kit (Molecular Probes, Invitrogen). In brief, exosome pellets were resuspended in 100 μl of 50 mM Tris-HCl (pH 8.0) and incubated with 100 μL of the Amplex Red reagent containing 2 U/mL HRP, 0.2 U/mL choline oxidase, and 100 μM acetylcholine in a final volume of 200 μL. Acetylcholinesterase and H_2_O_2_ solution were used as a positive control. The reaction plate was incubated in the absence of light for an hour at room temperature. Fluorescence measurement was taken every 15 min at a wavelength of 530 nm (λex) and 590 nm (λem), respectively.

### Animal treatment

In the present study, we used 10- to 12-week old C57BL/6 female mice. The animal study protocol was approved by the Institutional Animal Care and Use Committee (IACUC; approval number 18–086)) of the University of Tennessee Health Sciences Center (Memphis, TN, USA). All experiments were performed in accordance with relevant guidelines and regulations. Mice were divided into two groups, a control (n = 6) and a treatment group (n = 6). The control mice received Lieber-DeCarli control liquid diet and the treatment group mice received a single dose of ethanol (5 g kg^−1^). The animals were sacrificed at the end of the study and blood was collected to isolate plasma. Further, we isolated exosomes from plasma using a validated exosome isolation kit (Invitrogen, Life Technologies, NY) and characterized as described in the preliminary study and previous reports^28^.

### Exosomes labeling and uptake

After treating cells with isolated plasma exosomes, the cellular uptake of the exosomes was measured by using Exo-GLOW^TM^ Exosome Labeling Kits (System Biosciences, CA). Exosome pellets containing 100–500 µg protein were resuspended in 500 µl of 1X PBS. Next, 50 µl of 10X Exo-Green fluorescent label was added to the solution, mixed well by inversion, and incubated at 37 °C for 10 min. To this mixture, 100 µl of ExoQuick-TC reagent was added (mixed by inversion) to stop the labeling reaction and kept on ice (or at 4 °C) for 30 minutes. The samples were centrifuged at 16000 g for 3 minutes and the washed pellet was resuspended in 500 µl of 1X PBS. The labeled exosome suspensions were exposed to hepatocytes and their uptake was monitored by fluorescence microscopy.

### SiRNA transfection and treatment

We blocked CYP2E1 expression in HepaRG cells by transfecting the cells with predesigned human CYP2E1 siRNA or scrambled siRNA, followed by exosome treatment. Following 72-hour incubation with lipofectamine transfection reagent in serum-free and antibiotic-free media, siRNA-transfected cells were treated with plasma exosomes ± ALC and toxicity was compared with non-transfected treatment group to measure the specific contribution of plasma exosomal CYP2E1 in enhancing ALC-induced toxicity. Toxicity was measured by LDH assay.

### Western blot

Protein expression in the exosomes derived from human and murine plasma was determined by western blotting. Protein quantification was carried out by using a Pierce™ BCA protein assay (ThermoFisher Scientific, Grand Island, NY) as described before. Approximately 10 µg of protein was loaded into a polyacrylamide gel. The gel was run for 90 min at 150 V, which separated the proteins based on their molecular weight. To transfer the proteins from the gel to a polyvinyl fluoride membrane, gel was run for 90 minutes at 0.35 Amp. After the proteins were transferred to the membrane, it was blocked with 5–10 ml of Li-Cor blocking buffer (LI-COR Biosciences, Lincoln, NE) for 60 minutes to prevent the nonspecific binding of antibodies to its surface. The membrane was then incubated overnight with primary antibodies (CD63 rabbit Mab, 1:400 dilution, Proteintech, catalog #25682-1-AP; CD81 rabbit Mab 1:400 dilution, Santa Cruz Biotechnology, catalog #sc-9158; HP rabbit Mab, 1:500 dilution, Proteintech, catalog #16665-1-AP; CYP2E1 rabbit Mab, 1:400 dilution, Millipore, catalog #AB1252; SOD1 mouse Mab, 1:200 dilution, Santa Cruz Biotechnology, catalog #sc-101523; Catalase rabbit Mab, 1:400 dilution, Proteintech, catalog #21260-1-AP) at 4 °C. The membrane containing blots was washed with PBS containing 0.2% Tween-20 several times before incubating with the corresponding secondary antibodies for 1 h at room temperature, protected from light. The Image Studio Lite version 4.0 in a Li-Cor Scanner (LI-COR Biosciences) was used to scan the membrane blots. The fold change in the expression of proteins was calculated based on densitometry data obtained from the Image Studio Lite software.

### LDH cell viability assay

Cytotoxicity was measured using the Pierce Lactate Dehydrogenase (LDH) Cytotoxicity Assay Kit (ThermoFisher Scientific, Grand Island, NY) following the manufacturer’s protocol. Release of LDH into the cell culture media is an indicator of cytotoxicity and cytolysis. In brief, 50 μl of the collected media were mixed with 50 μl of the LDH reaction mixture in a 96-well plate. After incubating at room temperature for 30 minutes, the reaction was stopped by adding LDH stop solution. Using a micro plate reader (Cytation™ 5 Cell Imaging Multi-Mode Reader, BioTek, VT), the absorbance was measured at 490 nm and 680 nm. Higher absorbance indicated higher toxicity.

### Statistical analysis

Data analysis was carried out using GraphPad Prism 5 software (GraphPad Software Inc., San Diego, CA). All the data are presented as mean ± SEM of 3–6 experimental replicates. One-way ANOVA followed by Tukey’s post hoc test was used for comparisons between different treatment groups and p-values of ≤0.05 were considered statistically significant.

## Supplementary information

Dataset 1
