# Supplementary material for: Plasma exosomes exacerbate alcohol- and acetaminophen-induced toxicity via CYP2E1 pathway
Source: Sci Rep. 2019 Apr 25;9:6571. doi: 10.1038/s41598-019-43064-2 (PMC6484097; doi:10.1038/s41598-019-43064-2)
Supplement: Supplementary file 1 — Dataset 1 [file 41598_2019_43064_MOESM1_ESM.docx]

**Supplementary figure**

**Plasma exosomes exacerbate alcohol- and acetaminophen- induced toxicity via CYP2E1 pathway**

Mohammad A. Rahman^1^, Sunitha Kodidela^1^, Namita Sinha^1^, Sanjana Haque^1^, Pradeep K. Shukla^2^, Radhakrishna Rao^2^, and Santosh Kumar^1^*

^1^Department of Pharmaceutical Sciences, College of Pharmacy, University of Tennessee Health Science Center, Memphis, TN 38163 USA.

^2^Department of Physiology, University of Tennessee Health Science Center, Memphis, TN 38163 USA.

*****Author for correspondence:

 E-mail: [ksantosh@uthsc.edu](mailto:ksantosh@uthsc.edu); Tel: 901-448-7157

**Alcohol**

**Control**

**Protein; kDa**

**Supplementary figure 1: Western blot of CYP2E1, SOD1, and catalase proteins of plasma exosomes derived from binge-drinking mice model**. The number on each blot represent the number of samples in the respective groups.

**CD63; 65**

**CYP2E1; 57**

**SOD1; 16-18**

**Catalase; 55-60**


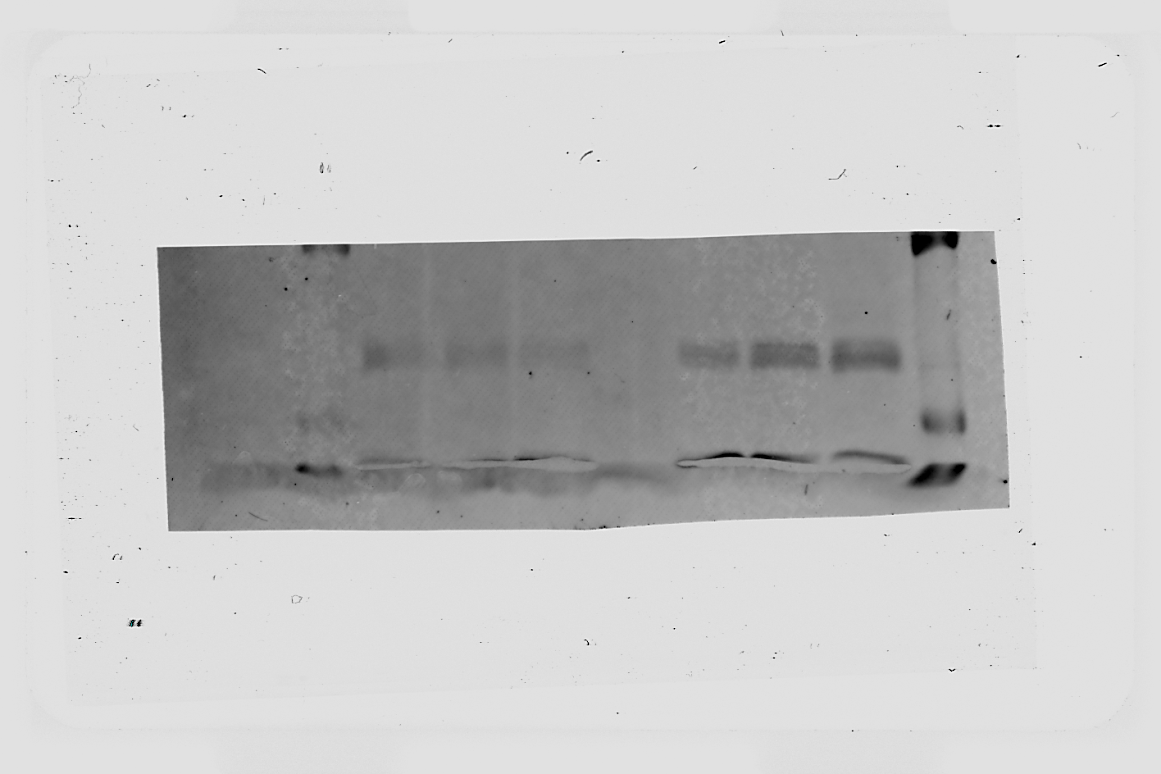


**1**

**2**

**3**

**1**

**2**

**3**


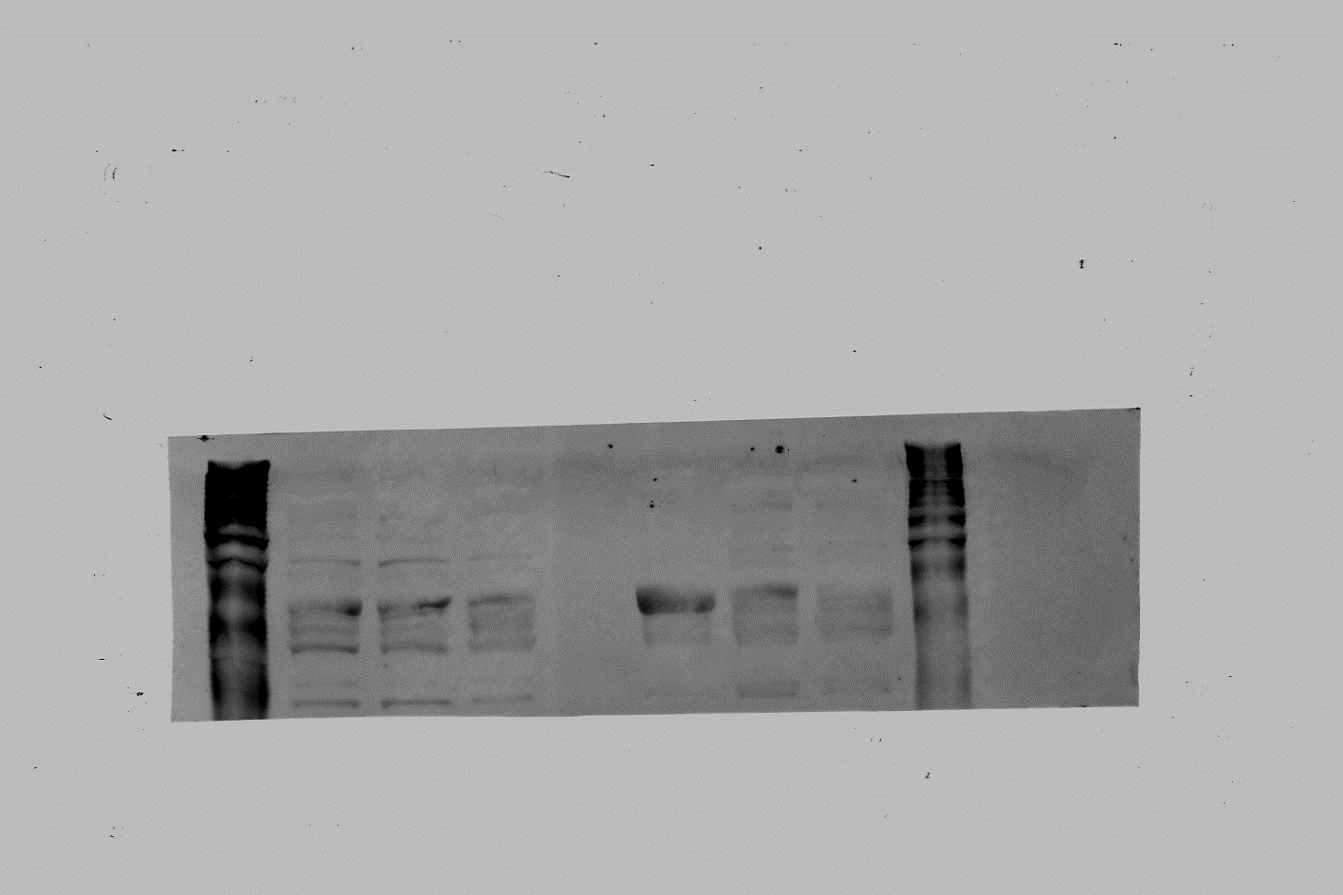


**1**

**2**

**3**

**1**

**2**

**3**


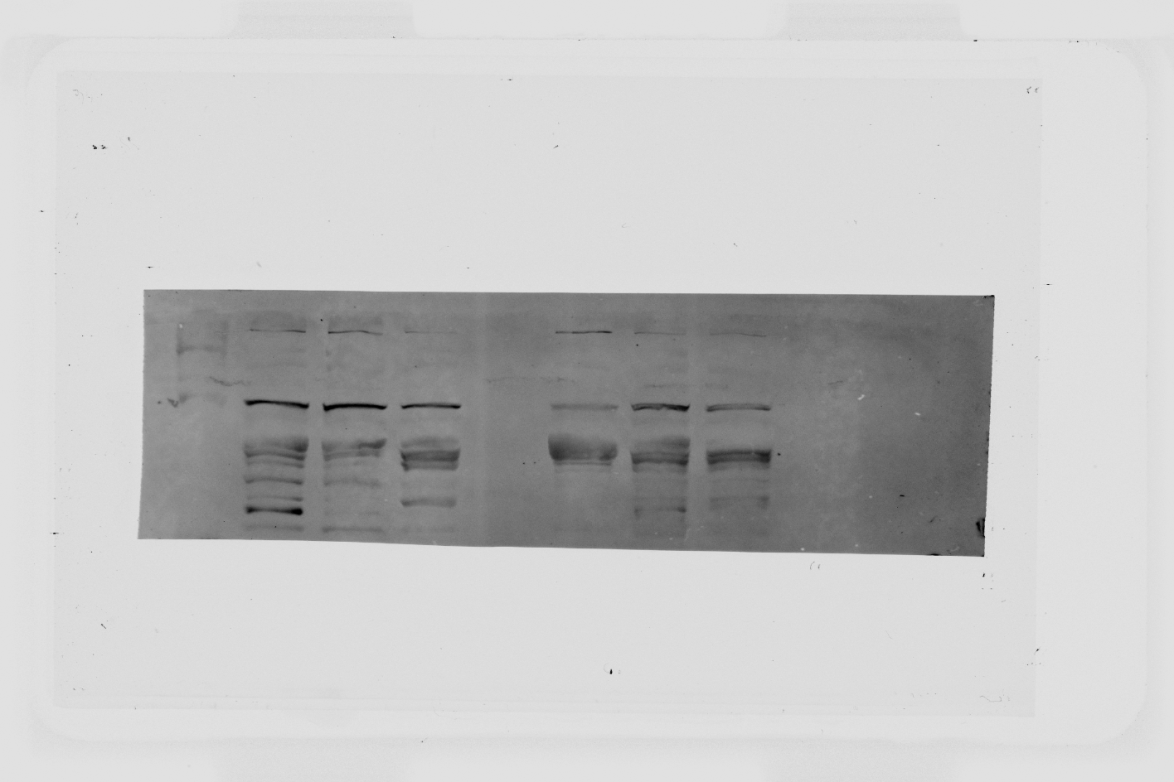


**1**

**2**

**3**

**1**

**2**

**3**


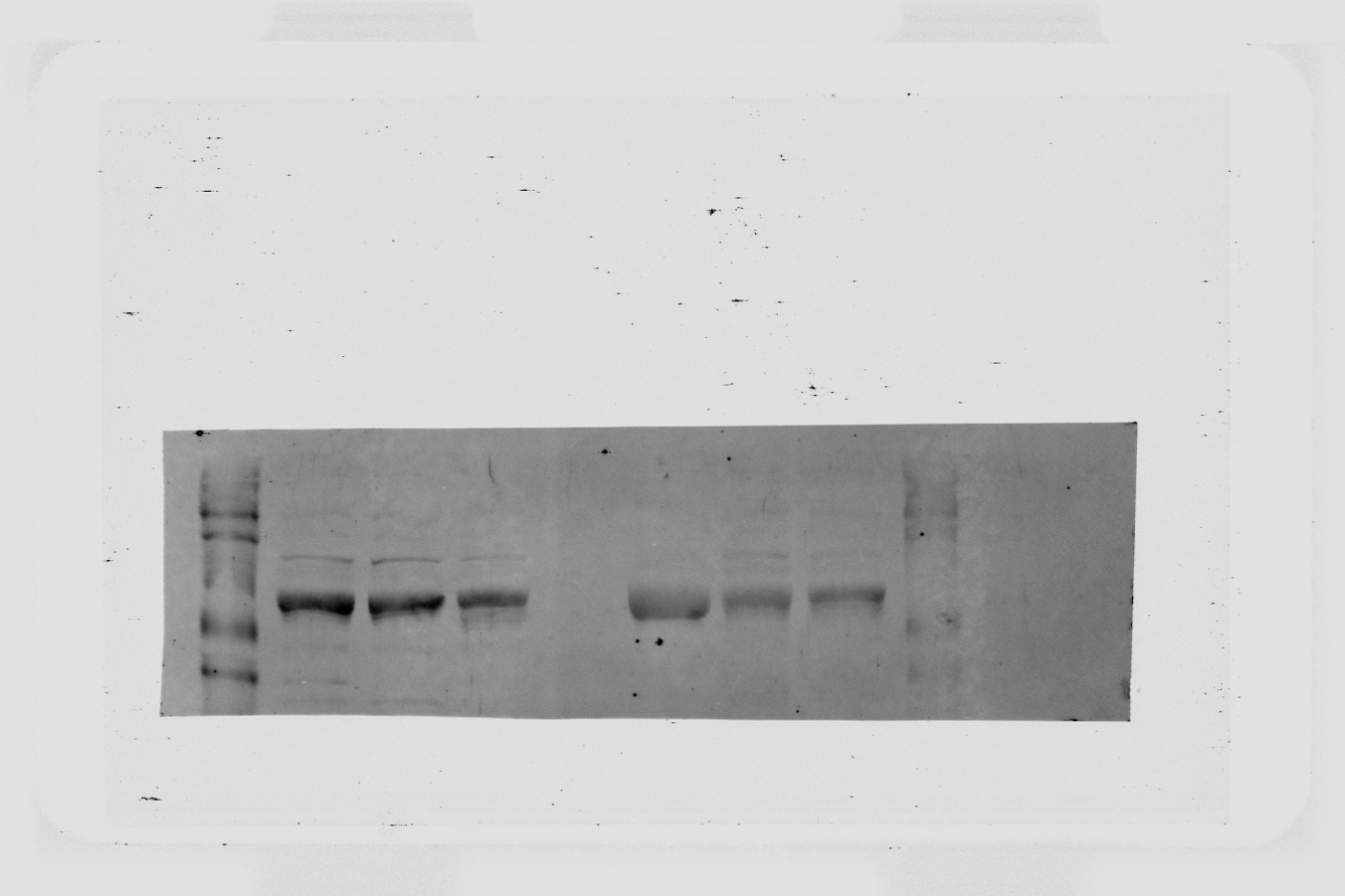


**1**

**2**

**3**

**1**

**2**

**3**
